# Successful Treatment of Facial Pseudolymphoma With Intravenous Rituximab: A Case Report

**DOI:** 10.1002/ccr3.71101

**Published:** 2025-10-01

**Authors:** Elham Behrangi, Nastaran Khodakarim, Shila Amiri, Seyyedeh Tahereh Rahimi, Neda Safarzadeh, Alireza Jafarzadeh

**Affiliations:** ^1^ Department of Dermatology, Hazrat Fatemeh Hospital, School of Medicine Iran University of Medical Sciences Tehran Iran; ^2^ Department of Medical Oncology and Hematology, Hazrat‐e Rasool General Hospital, School of Medicine Iran University of Medical Sciences Tehran Iran

**Keywords:** B‐cell pseudolymphoma, CD20, cutaneous pseudolymphoma, histopathology, immunohistochemistry, immunotherapy, lymphoproliferative disorder, refractory skin disease, rituximab, targeted therapy

## Abstract

Intravenous rituximab demonstrated significant efficacy in treating a case of refractory cutaneous pseudolymphoma that failed multiple conventional therapies, including corticosteroids, hydroxychloroquine, methotrexate, and biologics. The patient, a 42‐year‐old man, initially developed erythematous, nodular lesions on the face following dental implant surgery, which later spread and persisted despite various immunosuppressive and anti‐inflammatory treatments. Histopathological and immunohistochemical analysis confirmed B‐cell pseudolymphoma. Intravenous rituximab (500 mg weekly for 4 weeks, followed by maintenance doses) led to substantial lesion regression, with no recurrence observed after 1 year of follow‐up. This case underscores the therapeutic potential of rituximab for treatment‐resistant pseudolymphoma and highlights the need for further research into optimal treatment protocols for this rare condition.

**Abbreviations**
CLH: Cutaneous lymphoid hyperplasia
IHC: Immunohistochemistry
PML: Progressive multifocal leukoencephalopathy

**Summary**

- Intravenous rituximab demonstrated significant efficacy in treating refractory cutaneous pseudolymphoma, leading to substantial lesion regression after multiple failed therapies.
- This case highlights the potential of targeted B‐cell depletion in managing treatment‐resistant pseudolymphoma and underscores the need for further research into optimal therapeutic protocols.

## 1 Introduction

Pseudolymphoma is a benign lymphoproliferative disorder that closely resembles malignant lymphoma both clinically and histopathologically [1]. It arises as an exaggerated immune response to various external or internal stimuli, including tattoos, metal implants, contact allergens, vaccinations, certain medications, insect bites, and infections such as herpes zoster, Lyme disease, and borreliosis. Though it can affect individuals of all ages, its presentation and course can vary significantly [2, 3].

Cutaneous lymphoid hyperplasia (CLH), a form of pseudolymphoma, typically presents as erythematous, infiltrated plaques or nodules, usually measuring 1–3 cm in diameter, most commonly appearing on the head, neck, and upper extremities [4, 5]. The lesions may range from multiple clustered papules to larger nodules resembling panniculitis. Unlike some inflammatory dermatoses, pseudolymphoma lesions usually lack secondary changes such as scaling. The duration of the disease varies, with lesions persisting from weeks to several years [6].

Histopathologically, pseudolymphoma is characterized by dense dermal infiltration of lymphocytes, histiocytes, plasma cells, and eosinophils, forming nodular or diffuse patterns. The histological features often overlap with those of low‐grade B‐cell lymphomas, such as follicular center lymphoma and marginal zone lymphoma, or T‐cell lymphomas, including mycosis fungoides, lymphomatoid papulosis, cutaneous anaplastic large cell lymphoma, and subcutaneous panniculitis‐like T‐cell lymphoma. Immunohistochemical (IHC) staining is crucial for distinguishing pseudolymphoma from true lymphoma, as the pattern of lymphocytic infiltration and expression of specific markers guides accurate diagnosis [7, 8].

Due to its clinical and histological similarities to malignant lymphoma, diagnosing pseudolymphoma requires a comprehensive approach, incorporating clinical history, histopathological examination, and molecular studies. In some cases, pseudolymphoma may regress spontaneously or after biopsy, but persistent or symptomatic cases often require treatment. Management options include topical or intralesional corticosteroids, topical tacrolimus, simple excision, cryosurgery, laser ablation, photodynamic therapy, phototherapy, radiation therapy, hydroxychloroquine, and thalidomide [9, 10].

In refractory cases, targeted immunotherapy has emerged as a promising treatment strategy. Rituximab, a monoclonal antibody that selectively targets CD20‐positive B‐cells, induces apoptosis and effectively depletes abnormal lymphocytes. Recent studies have demonstrated the efficacy of rituximab in managing CD20‐positive pseudolymphoma, either through intralesional or intravenous administration [11, 12].

Previous reports have described the successful use of rituximab, either intralesionally or intravenously, in managing refractory cases of cutaneous pseudolymphoma. However, the existing evidence is limited to isolated case reports and small case series, highlighting the need for further documentation of treatment outcomes [1, 2, 3, 4].

This case report presents a patient with treatment‐resistant cutaneous pseudolymphoma who failed multiple therapeutic approaches, including corticosteroids, immunosuppressants, and biologics. The introduction of intravenous rituximab resulted in significant clinical improvement, highlighting its potential role in managing refractory cases of cutaneous pseudolymphoma.

## 2 Case History/Examination

A 42‐year‐old man presented with erythematous, nodular, infiltrative lesions that first appeared on his cheeks and the bridge of his nose following dental implant surgery. Over time, the lesions spread in a butterfly pattern to the lateral neck, shoulders, chest, and arms. They were mildly pruritic, non‐tender, and cosmetically concerning.

He promptly consulted a physician and was prescribed oral prednisolone (30 mg daily) along with antihistamines. After approximately 3 months, his symptoms were controlled, but residual lesions remained on his cheeks. A biopsy was performed, leading to a misdiagnosis of lupus erythematosus.

The patient was subsequently treated with hydroxychloroquine (200 mg daily) and prednisolone (20 mg daily). However, 1 week after initiating this regimen, he contracted COVID‐19 and discontinued all medications. During his 15‐day recovery at home, his facial lesions cleared. However, upon resuming outdoor activities, the lesions reappeared following sun exposure.

He restarted hydroxychloroquine (200 mg twice daily) and prednisolone (50 mg daily). This regimen was continued for several months, with prednisolone gradually tapered, but the lesions showed no improvement. He later presented to our clinic with erythematous, purple, pruritic infiltrative plaques on his face and was prescribed prednisolone (10 mg daily) and hydroxychloroquine (200 mg daily).

## 3 Methods

A biopsy was performed, with differential diagnoses including pseudolymphoma, tumid lupus, sarcoidosis, granuloma faciale, and granulomatous rosacea. The patient was treated with topical clobetasol, tacrolimus, and neotadine tablets. Due to a lack of response, intralesional triamcinolone (8 mg) was administered in multiple sessions.

**FIGURE 1:**
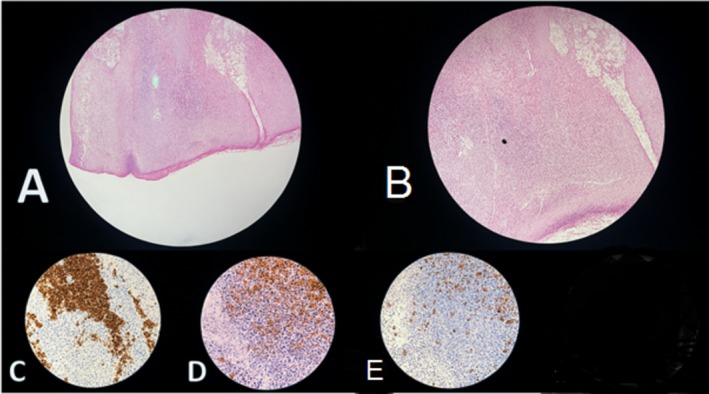
Histopathological and immunohistochemical features of cutaneous pseudolymphoma. Image A displays a low‐power (10×) H&E staining, revealing dense dermal lymphoid infiltration with follicular structures while maintaining epidermal integrity. Image B presents a high‐power (40×) H&E view, demonstrating a nodular lymphoid infiltrate composed of small lymphocytes, plasma cells, and scattered eosinophils. Image C illustrates CD20 immunostaining, confirming B‐cell predominance within the infiltrate. Image D shows CD3 immunostaining, highlighting interspersed T‐cells among the lymphoid clusters. Image E depicts kappa and lambda light chain immunostaining, indicating a polyclonal B‐cell population and ruling out monoclonality. These combined findings support the diagnosis of cutaneous pseudolymphoma over malignant lymphoma.

Histopathological examination of a skin biopsy from the lesion revealed dense dermal infiltration composed predominantly of small‐sized lymphocytes and histiocytes, interspersed with scattered eosinophils forming multiple follicles with germinal centers. Increased vascularity, eosinophilic infiltration, and tingible body macrophages were also observed (Figure 1).

The overlying epidermis showed focal parakeratosis, acanthosis, and mild spongiosis. Immunohistochemical staining confirmed a B‐cell pseudolymphoma without malignant features.

In addition to histopathological examination and immunohistochemical profiling, an extended diagnostic work‐up was undertaken to rigorously exclude a malignant lymphoproliferative disorder. Evaluation of light chain restriction via kappa and lambda immunostaining revealed a balanced kappa/lambda ratio, indicative of a polyclonal B‐cell population. To further substantiate this finding, molecular analysis was conducted using polymerase chain reaction (PCR) targeting immunoglobulin heavy chain (IGH) gene rearrangements. The PCR assay yielded a polyclonal pattern without evidence of monoclonal peaks, effectively ruling out clonal B‐cell proliferation. This comprehensive approach ensured a high level of diagnostic certainty, differentiating cutaneous pseudolymphoma from primary cutaneous B‐cell lymphomas and other malignancies.

Based on the integration of clinical presentation, histopathological examination, immunohistochemical profiling, kappa/lambda light chain analysis, and PCR assessment for IGH gene rearrangements, a definitive diagnosis of cutaneous pseudolymphoma was established.

Given the patient's photosensitivity, prior transient response to hydroxychloroquine, and initial misdiagnosis, tumid lupus was thoroughly considered in the differential diagnosis. To address this, repeat histopathological assessments were conducted, which consistently lacked dermal mucin deposition, absence of interface changes, and no evidence of basal layer vacuolization—hallmark features of lupus erythematosus. In addition, direct immunofluorescence (DIF) on perilesional skin was negative for IgG, IgM, IgA, and C3 deposition at the dermoepidermal junction. Comprehensive serologic evaluation including antinuclear antibody (ANA), anti‐dsDNA, anti‐Ro/SSA, and anti‐La/SSB antibodies yielded negative results. This combined histopathological, immunopathological, and serological profile effectively ruled out tumid lupus and reinforced the diagnosis of cutaneous pseudolymphoma with a high degree of diagnostic certainty.

The patient underwent cryotherapy, which initially led to a reduction in lesion induration, but the lesions persisted. He was subsequently started on mycophenolate mofetil (CellCept) 500 mg every 6 h, and laboratory tests were conducted to assess eligibility for baricitinib. Once all lab results were within normal limits, baricitinib (4 mg daily) was introduced.

Despite these treatments, the patient did not respond, prompting a repeat biopsy, which again confirmed pseudolymphoma. Weekly methotrexate (10 mg) was then initiated alongside hydroxychloroquine (200 mg twice daily) and three betamethasone LA injections over 3 weeks. Due to a lack of improvement and lesion progression, the methotrexate dose was increased to 15 mg weekly; however, the patient still did not respond.

The patient was later admitted to the hospital and started on intravenous rituximab (500 mg weekly for 4 weeks). Two additional rituximab injections were administered at four‐month intervals.

Prior to rituximab initiation, comprehensive screening was conducted including:

- Hepatitis B virus (HBV) screening: HBsAg, anti‐HBc, and HBV DNA all negative.
- Tuberculosis screening: Interferon‐Gamma Release Assay (IGRA, QuantiFERON‐TB Gold) was negative.

Peripheral B‐cell counts were evaluated using flow cytometry (CD19+ and CD20+ markers) 1 month after induction therapy, confirming effective depletion (< 1% CD19+ cells).

Serum immunoglobulin levels (IgG, IgA, and IgM) were measured at baseline, 3, 6, and 12 months post‐treatment, and remained within the normal range.

Routine laboratory tests, including complete blood count (CBC), renal and liver function tests, and serum protein electrophoresis, were monitored regularly during follow‐up.

## 4 Conclusion and Results

**FIGURE 2:**
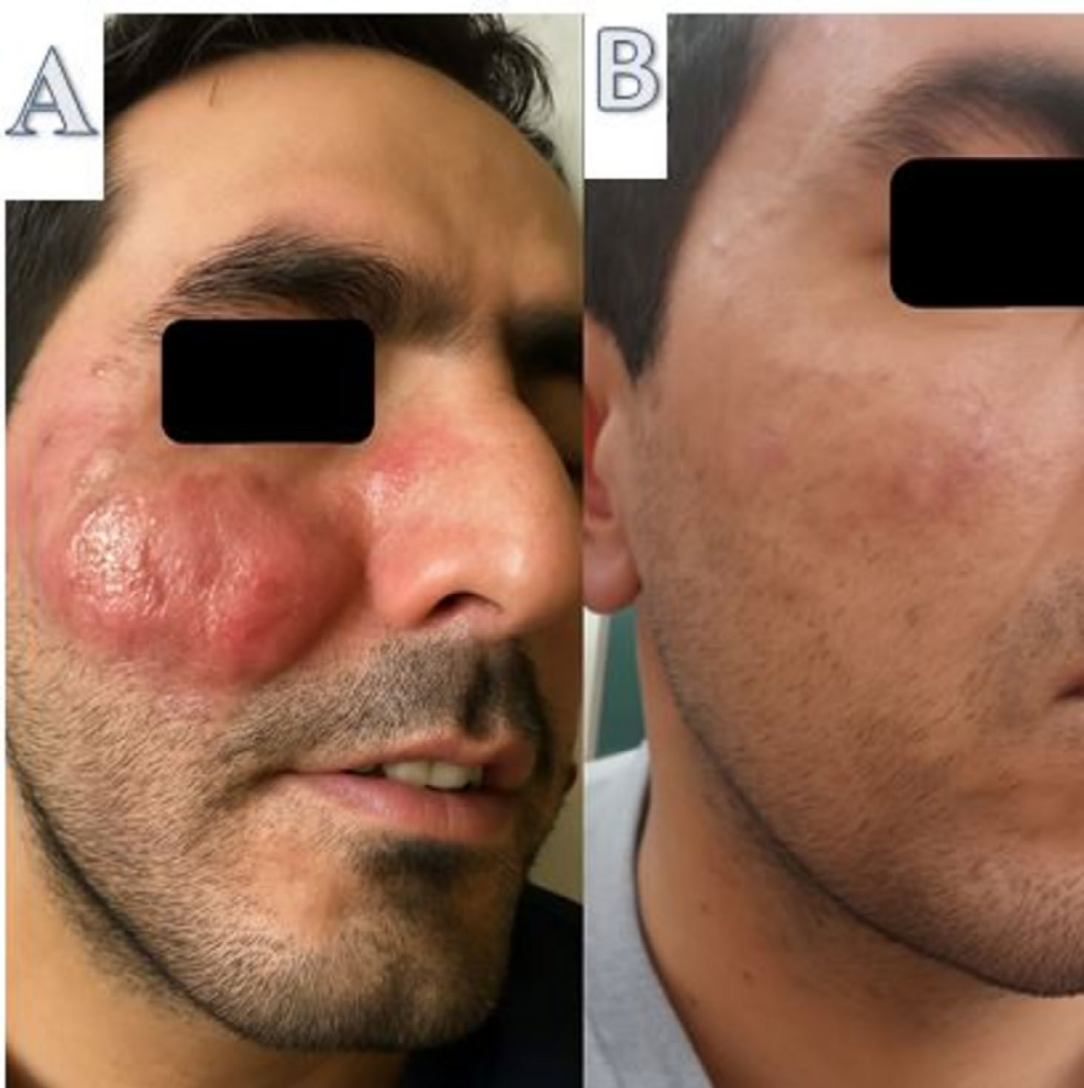
Significant healing of facial lesions following treatment with Rituximab. (A) Before, (B) After.

The introduction of intravenous rituximab (500 mg weekly for 4 weeks) led to a marked reduction in lesion size and erythema after the second infusion. Two additional rituximab injections were administered at four‐month intervals, resulting in significant disease regression. By the end of the 4‐week course, the lesions had significantly diminished, and no recurrence was observed during 1 year of follow‐up (Figure 2).

The patient's peripheral B‐cell population was effectively depleted following rituximab induction, as confirmed by CD19+ and CD20+ flow cytometry showing < 1% B‐cells at 1 month. Immunoglobulin levels remained stable across all timepoints up to 12 months post‐treatment. Additionally, HBV reactivation screening and TB surveillance remained negative, and no laboratory abnormalities or infections occurred during the monitoring period.

This case highlights the challenges in diagnosing and managing cutaneous pseudolymphoma, particularly in patients with treatment‐resistant disease. The patient's condition remained refractory to multiple immunosuppressive and anti‐inflammatory therapies, including corticosteroids, antimalarials, immunomodulators, and biologics. However, intravenous rituximab demonstrated substantial efficacy, leading to a significant reduction in lesion burden. These findings suggest that rituximab may be a viable treatment option for refractory cases of cutaneous pseudolymphoma. Regular follow‐up is essential to monitor disease recurrence and assess long‐term treatment outcomes.

This case adds to the limited but expanding clinical experience with rituximab in cutaneous pseudolymphoma. Future studies with larger cohorts and longer follow‐up periods are necessary to establish optimal dosing regimens and to evaluate the durability of treatment response.

## 5 Discussion

Cutaneous pseudolymphoma represents a challenging diagnostic and therapeutic entity due to its clinical and histopathological resemblance to malignant lymphoma [13]. Differentiating pseudolymphoma from true lymphoma requires a comprehensive approach incorporating histopathological, immunohistochemical, and molecular analyses. Despite its benign nature, persistent and treatment‐resistant cases often necessitate systemic immunosuppressive or targeted therapies [1, 3].

In recent years, several reports have described the use of intravenous rituximab in treatment‐refractory cutaneous pseudolymphoma, with encouraging results. Balode et al. [2] reported a case of T‐cell pseudolymphoma treated successfully with weekly IV rituximab after failure of steroids and topical therapies. Similarly, Besch‐Stokes et al. [1] described a 72‐year‐old man with B‐cell pseudolymphoma who responded dramatically to weekly rituximab infusions, followed by methotrexate maintenance. A more recent 2024 case by Suwanchatkul et al. used a rheumatoid arthritis–based rituximab regimen (1 g IV on Days 1 and 15) with full remission and successful retreatment upon relapse. These studies highlight the emerging role of rituximab in managing resistant cases [14].

In our case, an extensive diagnostic work‐up was undertaken beyond standard histopathology and immunohistochemistry (IHC). Light chain restriction was evaluated via kappa and lambda immunostaining, which demonstrated a balanced expression pattern consistent with polyclonal B‐cell proliferation. Furthermore, polymerase chain reaction (PCR) analysis for immunoglobulin heavy chain (IGH) gene rearrangements confirmed a polyclonal profile, with no evidence of monoclonality. This comprehensive diagnostic strategy provided robust confirmation of pseudolymphoma and reliably excluded a malignant lymphoproliferative disorder.

Furthermore, the possibility of tumid lupus was rigorously excluded based on clinical‐pathologic correlation. Histological evaluations showed no mucin deposition or interface dermatitis; direct immunofluorescence was negative, and an extensive autoantibody panel returned negative. These cumulative findings provided diagnostic clarity, differentiating pseudolymphoma from autoimmune mimickers such as tumid lupus.

The patient presented with a chronic, treatment‐refractory form of cutaneous pseudolymphoma that was unresponsive to multiple conventional therapies, including corticosteroids, hydroxychloroquine, methotrexate, mycophenolate mofetil, and biologics such as baricitinib. The decision to initiate intravenous rituximab was guided by emerging evidence supporting its efficacy in CD20‐positive B‐cell lymphoproliferative disorders, including primary cutaneous B‐cell lymphomas and refractory pseudolymphomas.

Rituximab, a chimeric monoclonal antibody targeting CD20‐positive B‐cells, plays a pivotal role in depleting aberrant lymphocytes while preserving long‐term immune function [2]. Its mechanism of action includes antibody‐dependent cellular cytotoxicity, complement‐mediated lysis, and direct apoptosis induction. The dramatic improvement observed in our patient following rituximab therapy underscores its potential as a targeted treatment option for resistant cases of pseudolymphoma [4].

Several case reports and small‐scale studies have demonstrated the efficacy of rituximab in cutaneous pseudolymphoma, particularly in cases that fail standard therapies. Previous studies have reported success with both intralesional and systemic administration of rituximab, leading to significant lesion regression [2, 3, 4]. In our case, the intravenous regimen (500 mg weekly for 4 weeks, followed by maintenance doses at four‐month intervals) resulted in substantial lesion reduction and sustained remission for over a year. This suggests that intravenous rituximab may provide a durable response in refractory pseudolymphoma cases.

Despite its promising efficacy, rituximab therapy carries potential risks, including infusion reactions, infections due to B‐cell depletion, and rare instances of progressive multifocal leukoencephalopathy (PML) [7]. Close monitoring and pretreatment screening for infections such as hepatitis B and tuberculosis are essential to minimize complications [10]. In our patient, no significant adverse effects were observed, and laboratory parameters remained stable throughout treatment.

This case highlights the importance of considering rituximab in refractory cutaneous pseudolymphoma, particularly in cases unresponsive to conventional therapies. Future studies, including larger cohort analyzes and long‐term follow‐ups, are necessary to establish standardized treatment protocols and identify optimal dosing regimens. Additionally, investigating predictive biomarkers for treatment response could further refine patient selection and improve therapeutic outcomes.

While several case reports and small‐scale studies have reported favorable responses to rituximab in treating cutaneous pseudolymphoma, no standardized treatment protocols exist. Our case further contributes to the growing body of evidence supporting the use of intravenous rituximab in treatment‐refractory cases.

Consistent with these reports, our patient experienced a marked and sustained clinical response to systemic rituximab, despite resistance to multiple prior immunosuppressive regimens. The absence of adverse effects and the prolonged remission observed in our case support the expanding evidence that rituximab is not only effective but also well‐tolerated in select patients with cutaneous pseudolymphoma.

In conclusion, intravenous rituximab demonstrated remarkable efficacy in our patient, leading to significant clinical improvement and sustained remission. This case reinforces the potential of targeted B‐cell depletion therapy in managing resistant pseudolymphoma and highlights the need for further research to optimize treatment strategies for this challenging condition.

## Author Contributions

**Elham Behrangi:** conceptualization, data curation, project administration. **Nastaran Khodakarim:** investigation, validation, writing – review and editing. **Shila Amiri:** investigation, methodology. **Seyyedeh Tahereh Rahimi:** visualization, writing – original draft. **Neda Safarzadeh:** validation, visualization. **Alireza Jafarzadeh:** conceptualization, project administration.

## Disclosure

Transparency Declaration: Authors declare that the manuscript is honest, accurate, and transparent. No important aspect of the study is omitted.

## Ethics Statement

The researchers were committed and adhered to the principles of the Helsinki Convention and the Ethics Committee of the Iran University of Medical Sciences in all stages.

## Consent

After providing the necessary explanations, written informed consent was obtained from the patient regarding the submission of their clinical condition to medical journals. Additionally, the patient has been assured that their name and personal details will be kept confidential by the authors.

## Data Availability

All data produced in the present study is available upon reasonable request to the authors.
